# Editorial: Allergens and allergic sensitization in Asia and the tropics—volume II

**DOI:** 10.3389/falgy.2026.1973136

**Published:** 2026-09-18

**Authors:** Kyoung Yong Jeong, Alain Jacquet, Ting Fan Leung, Philip Hei Li

**Affiliations:** 1 Department of Internal Medicine, Institute of Allergy, Yonsei University College of Medicine, Seoul, Republic of Korea; 2 Department of Biochemistry, Faculty of Medicine, Chulalongkorn University, Bangkok, Thailand; 3 Department of Paediatrics, Faculty of Medicine, The Chinese University of Hong Kong, Shatin, Hong Kong SAR, China; 4 School of Clinical Medicine, Li Ka Shing Faculty of Medicine, The University of Hong Kong, Pokfulam, Hong Kong SAR, China

**Keywords:** allergic sensitization, allergy immunotherapy, Asia, environmental factors, ige cross-reactivity, molecular allergen profiling, tropical allergens, tropics

The prevalence of allergic diseases has dramatically increased in the Asia-Pacific region (1, 2). This rapid rise may be attributed to global warming, extensive urbanization, associated environmental pollution, and improved sanitation (3).

Accurate allergy diagnostics and immunotherapeutic approaches should account for regional differences in sensitization patterns (4). However, some allergens in Eastern countries have not yet been fully characterized and most of the commercial diagnostic tools are focused on western allergic sensitizations. Geographical specificities can be exemplified by cedar pollen, the main cause of hay fever in Japan, and *Blomia tropicalis*, the predominant mite species in tropical regions, whereas sensitization to birch pollen and *Dermatophagoides pteronyssinus* is more prevalent in Western countries. Moreover, certain foods consumed primarily in Asia may contribute to the development of region-specific food allergies. For instance, horse milk allergy is relatively common in Mongolia, along with lactose intolerance. Horse milk is often used as a substitute for children with cow's milk allergy, and fermented mare's milk (airag) is a national staple (personal communication with Prof. Munhbayarlah at the National University of Mongolia). Some important allergens from fruits and vegetables that elicit pollen-food allergy syndrome in Korea have yet to be characterized (5). Individuals may also develop allergies to foods encountered for the first time in a foreign country or to imported products as a result of cross-reactivity with pollen allergens from their country of origin. Interestingly, allergenic components can also be found in traditional and herbal medicines. For example, Pana g 1, a ginseng allergen homologous to Bet v 1, has been detected in some ginseng beverages (6). Japanese hop is also widespread in far east Asian countries, while it is now invasive in western countries (7). Therefore, it is important to investigate the similarities and differences between these regional particularities and those observed in Western and tropical countries.

The aim of this Research Topic is to provide an update on patterns of allergic sensitization in Asian and tropical countries, with an emphasis on their similarities to and differences from those observed in Western industrialized countries. This Research Topic serves as an expanded version of a previous Research Topic (8). In the current topic, five articles were collected, reflecting the rapid growth of knowledge and the critical need to characterize regionally important allergens. Two articles focused on sensitization patterns in eastern China, while three studies addressed molecular diagnosis in Thailand, Colombia, and India. In a retrospective analysis from eastern China, *D. pteronyssinus* (37.58%) was the most common respiratory allergen source, followed by mold (13.11%). Cashew nut (15.57%) was the most frequently identified food allergen, followed by cow's milk (10.99%) and egg (9.33%). These findings represent a distinct sensitization pattern that may reflect regional, lifestyle and cultural differences. Huang et al. investigated differential food sensitization patterns among infants, toddlers, and children. Their results suggest that sensitization patterns emerge early in life, highlighting the need for differentiated allergy risk management and prevention strategies across different developmental stages. Kulalert et al. reported better diagnostic performance of specific IgE (sIgE) measurement for shrimp tropomyosin than that of the skin prick test and sIgE measurement using shrimp extract. Marrugo et al. demonstrated that molecular diagnosis using combinations of specific allergens can improve diagnostic accuracy in Colombia, potentially supporting personalized allergy management. Ghosh et al. evaluated the diagnostic value of recombinant Asp f 10, a secreted aspartic protease, and Asp f 10-specific IgG as a biomarker for allergic bronchopulmonary aspergillosis.

A rapidly growing body of knowledge is evident regarding novel allergens from Asian and tropical regions. Certain sensitization patterns may be specific to these geographic areas, which encompass temperate to tropical climates and harbor diverse endemic flora and fauna. Although many of these allergens can exhibit cross-reactivity with allergens from similar sources in other regions, the integration of such “tropical/Asia”-specific components will improve the diagnosis of allergic sensitization in these regions of the world.

## References

[1] Leung AS Pacharn P Tangvalelerd S Sato S Pitt E Wong G. Food allergy in a changing dietary landscape: a focus on the Asia Pacific region. Pediatr Allergy Immunol. (2024) 35(8):e14211. 10.1111/pai.14211 39127915

[2] Yin X Zhang X Xu F Xiao H Meng J Li PH. Landscape of aeroallergen sensitization in Asia. Clin Exp Allergy. (2025) 55(10):901–15. 10.1111/cea.70137 40820585 PMC12515540

[3] Agache I Akdis C Akdis M Al-Hemoud A Annesi-Maesano I Balmes J. Climate change and allergic diseases: a scoping review. J Clim Chang Health. (2024) 20:100350. 10.1016/j.joclim.2024.100350 41647117 PMC12851229

[4] Riggioni C Leung AS Wai CY Davies JM Sompornrattanaphan M Pacharn P. Exploring geographical variances in component-resolved diagnosis within the Asia-Pacific region. Pediatr Allergy Immunol. (2025) 36(3):e70054. 10.1111/pai.70054 40105317 PMC11921761

[5] Jeong KY Park JW. Neglected but clinically relevant allergens in Korea. Curr Allergy Asthma Rep. (2024) 24(9):519–26. 10.1007/s11882-024-01161-x 38980649

[6] Jeong KY Shin YJ Kim H Lee YS Sang M Oh HK. Characterization of Pana g 1, an important cause of pollen-food allergy syndrome from Korean ginseng, *Panax ginseng*. World Allergy Organ J. (2026) 19(1):101164. 10.1016/j.waojou.2025.101164 41550676 PMC12805375

[7] Jeong KY Sang M Lee YS Gadermaier G Ferreira F Park JW. Characterization of hum j 6, a major allergen from Humulus japonicus pollen, the primary cause of weed pollinosis in east Asia. Allergy, Asthma Immunol Res. (2023) 15(6):767–78. 10.4168/aair.2023.15.6.767 37957794 PMC10643856

[8] Caraballo L. Editorial: allergens and allergic sensitization in Asia and the tropics. Front Allergy. (2021) 2:808044. 10.3389/falgy.2021.808044 35386982 PMC8974715

